# New Measles Genotype, Uganda

**DOI:** 10.3201/eid1110.050431

**Published:** 2005-10

**Authors:** Apollo Muwonge, Miriam Nanyunja, Paul A. Rota, Josephine Bwogi, Luis Lowe, Stephanie L. Liffick, William J. Bellini, Sempala Sylvester

**Affiliations:** * Uganda Virus Research Institute, Entebbe, Uganda; † World Health Organization, Kampala, Uganda; ‡ Centers for Disease Control and Prevention, Atlanta, Georgia, USA

**Keywords:** Measles, virus, isolation, sequencing, genotype, research

## Abstract

We report the first genetic characterization of wildtype measles viruses from Uganda. Thirty-six virus isolates from outbreaks in 6 districts were analyzed from 2000 to 2002. Analyses of sequences of the nucleoprotein (N) and hemagglutinin (H) genes showed that the Ugandan isolates were all closely related, and phylogenetic analysis indicated that these viruses were members of a unique group within clade D. Sequences of the Ugandan viruses were not closely related to any of the World Health Organization reference sequences representing the 22 currently recognized genotypes. The minimum nucleotide divergence between the Ugandan viruses and the most closely related reference strain, genotype D2, was 3.1% for the N gene and 2.6% for the H gene. Therefore, Ugandan viruses should be considered a new, proposed genotype (d10). This new sequence information will expand the utility of molecular epidemiologic techniques for describing measles transmission patterns in eastern Africa.

## Abstract 2

New measles virus genotype will increase epidemiologic and virologic surveillance in Africa.

Measles virus is a negative-sense, single-stranded RNA virus in the genus Morbillivirus within the family Paramyxoviridae. Infection with this virus is typified by high fever, maculopapular rash, conjunctivitis, cough, and coryza (1). Although a vaccine-preventable disease, it still accounts for ≈770,000 deaths annually worldwide, half of which occur in Africa (2).

This virus is monotypic, but genetic variation in the hemagglutinin (H) and nucleoprotein (N) genes can be analyzed by molecular epidemiologic techniques to study transmission patterns. This molecular information, in conjunction with standard case reporting and investigation, is useful in assessing the effectiveness of vaccination programs (*3**–**5*). Genetic characterization of wildtype measles virus is a key component of laboratory surveillance activities in all phases of measles control. To facilitate genetic characterization of measles viruses, a uniform nomenclature and analysis protocol was recommended by the World Health Organization (WHO) (*6*). WHO currently recognizes 22 genotypes of measles virus and has established guidelines for the designation of new genotypes (*5**–**8*).

In Uganda, measles is still endemic despite the availability of a measles vaccine for almost 2 decades. This failure to completely control measles is mainly the result of inadequate coverage of measles immunization, which is a single dose of vaccine given at 9 months of age. The reported measles vaccination coverage rate in 2002 was only 74%. Given a seroconversion rate of ≈85% for measles vaccine given at 9 months of age, ≈37% of Ugandan children are expected to remain susceptible to measles. The measles vaccination coverage increased to 83% in 2003 and 2004. The number of measles cases reported to the Ugandan National Health Management Information System ranged from 57,347 in 1997 to 49,871 in 2002. Of the cases reported in 2002, 31% occurred in persons >5 years of age. The Ministry of Health developed a 5-year plan (2002–2006) aimed at reducing the illness and death caused by measles virus. The strategies include increasing routine immunization coverage, conducting vaccination campaigns for children 6 months to 15 years of age, providing vitamin A, and initiating a case-based measles surveillance system. In October 2003, Uganda conducted a catch-up campaign for children 6 months to 15 years of age that reached ≈13.5 million children with measles vaccine, with a national coverage of 105%. This campaign resulted in a decrease in reported measles cases to 3,522 from January to August 2004, compared with 28,072 in 2003 and 33,633 in 2002 in the same period, with no deaths among confirmed measles cases as of August 2004. Case-based measles surveillance was established in 2003, and by August 2004, >80% of districts were investigating measles cases according to the guidelines.

Some information is available on circulating measles virus genotypes in western, central, and southern Africa, but little is known about circulating strains in eastern Africa, including Uganda. The WHO Regional Measles Laboratory at Uganda Virus Research Institute (UVRI) undertook the present study to isolate and characterize circulating measles strains in Uganda.

## Materials and Methods

### Specimen Collection and Virus Isolation

Specimens for virus isolation were collected from outbreaks in different districts of Uganda, sentinel sites, and Mulago hospital, the national teaching and referral hospital, in Kampala from 2000 to 2002. Staff members from the UVRI were part of the outbreak investigation team and responsible for collecting specimens. Urine and nasopharyngeal aspirate specimens were obtained <7 days of onset of a rash according to WHO procedures for laboratory diagnosis of measles viral infection (*9*) and transported to UVRI for processing by using standard procedures.

The specimens were added onto B95a cells (*10*) that had been seeded into 25-cm^2^ tissue culture flasks and observed daily for characteristic cytopathic effect (CPE). Infected cells were harvested when >75% of the culture showed CPE, and viral stocks were prepared and stored at –70°C in 0.5-mL aliquots. Specimens contaminated with bacteria or fungi were filtered through a 0.45-μm filter, and tissue culture additions were repeated. Isolates were shipped to the Centers for Disease Control and Prevention (CDC) in Atlanta, Georgia, for molecular analysis.

### Polymerase Chain Reaction and Sequencing

At CDC, the isolates were passaged once in B95a cells. RNA was extracted from infected cells by using the guanidinium acid-phenol technique (*11*), and reverse transcriptase-polymerase chain reaction (RT-PCR) was used to amplify either the 550 nucleotides (nt) coding for the COOH terminus of N or the full-length open reading frame for H (*12*). PCR products were purified by using the PCR Preps DNA Purification System (Promega, Madison, WI, USA) and analyzed by agarose gel electrophoresis followed by staining with ethidium bromide. Templates were sequenced by using a cycle sequencing reaction with fluorescent dye terminators (Perkin-Elmer, Applied Biosystems Division, Foster City, CA, USA), and the reaction products were analyzed by using an ABI 3100 (Perkin-Elmer) automatic sequencer. Sequence data from multiple reactions were analyzed with version 10.1 of the Genetics Computer Group Package (Accelrys, San Diego, CA, USA). Phylogenetic analyses, including bootstrap analysis, were performed by using PAUP version 4.01 (Sinauer Associates, Sunderland, MA, USA). The sequence of the 450 nt coding for the 150 amino acids at the COOH terminus of the N gene was obtained for all the isolates, and the entire coding region of the H gene was sequenced for 2 representative isolates.

## Results

**Figure 1:**
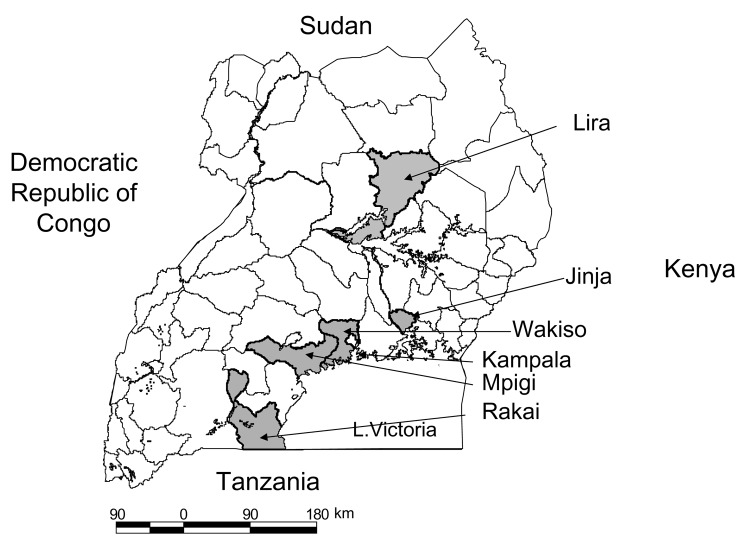
Map of Uganda showing districts where measles virus isolates were obtained from 2000 to 2002.

Thirty-six measles virus isolates obtained from measles outbreaks (n = 8) and Mulago Hospital (n = 28) from 2000 to 2002 were selected for genetic analysis. Isolates were obtained from 6 districts within Uganda (Table 1 and Figure 1), although most were obtained from patients in Kampala. Viruses were isolated from either urine or respiratory samples collected within 7 days of rash onset. Overall, virus isolations were successful in 14% of the specimens.

**Table 1:** Wildtype measles viruses isolated in Uganda, 2000–2002*

WHO name and genotype	Collection date
MVi/Kampala UGA/10.00-1[d10]†	3/5/2000
MVi/Kampala UGA/10.00-2[d10]	3/6/2000
MVi/Mpigi UGA/18.00[d10]†	4/27/2000
MVi/Kampala UGA/42.00-1[d10]	10/10/2000
MVi/Kampala UGA/42.00-2[d10]	10/16/2000
MVi/Kampala UGA/43.00-1[d10]	10/23/2000
MVi/Kampala UGA/43.00-2[d10]	10/23/2000
MVi/Kampala UGA/45.00[d10]	11/6/2000
MVi/Kampala UGA/46.00-1[d10]	11/8/2000
MVi/Kampala UGA/46.00-2[d10]	11/13/2000
MVi/Kampala UGA/49.00-1[d10]	11/28/2000
MVi/Kampala UGA/49.00-2[d10]	11/30/2000
MVi/Kampala UGA/50.00[d10]	12/11/2000
MVi/Kampala UGA/51.00-1[d10]‡	12/12/2000
MVi/Kampala UGA/51.00-2[d10]	12/18/2000
MVi/Kampala UGA/51.00-3[d10]†	12/18/2000
MVi/Kampala UGA/51.00-4[d10]	12/18/2000
MVi/Kampala UGA/3.01[d10]	1/15/2001
MVi/Kampala UGA/4.01-1[d10]†	1/24/2001
MVi/Kampala UGA/4.01-2[d10]	1/24/2001
MVi/Kampala UGA/6.01[d10]	2/8/2001
MVi/Kampala UGA/8.01[d10]	2/22/2001
MVi/Kampala UGA/9.01[d10]	2/27/2001
MVi/Lira UGA/12.01[d10]	3/19/2001
MVi/Kampala UGA/12.01[d10]	3/25/2001
MVi/Kampala UGA/15.01-1[d10]	4/9/2001
MVi/Kampala UGA/15.01-2[d10]	4/10/2001
MVi/Kampala UGA/15.01-3[d10]	4/11/2001
MVi/Rakai UGA/17.01[d10]	4/23/2001
MVi/Mpigi UGA/18.01-1[d10]	5/4/2001
MVi/Mpigi UGA/18.01-2[d10]	5/4/2001
MVi/Mpigi UGA/18.01-3[d10]	5/5/2001
MVi/Kampala UGA/32.01-1[d10]	8/7/2001
MVi/Kampala UGA/32.01-2[d10]	8/7/2001
MVi/Wakiso UGA/32.01[d10]	8/8/2001
MVi/Jinja UGA/8.02[d10]†	2/24/2002

*WHO, World Health Organization. †Members of the second, smaller cluster of Ugandan viruses. ‡Reference strain for proposed genotype d10.

**Figure 2:**
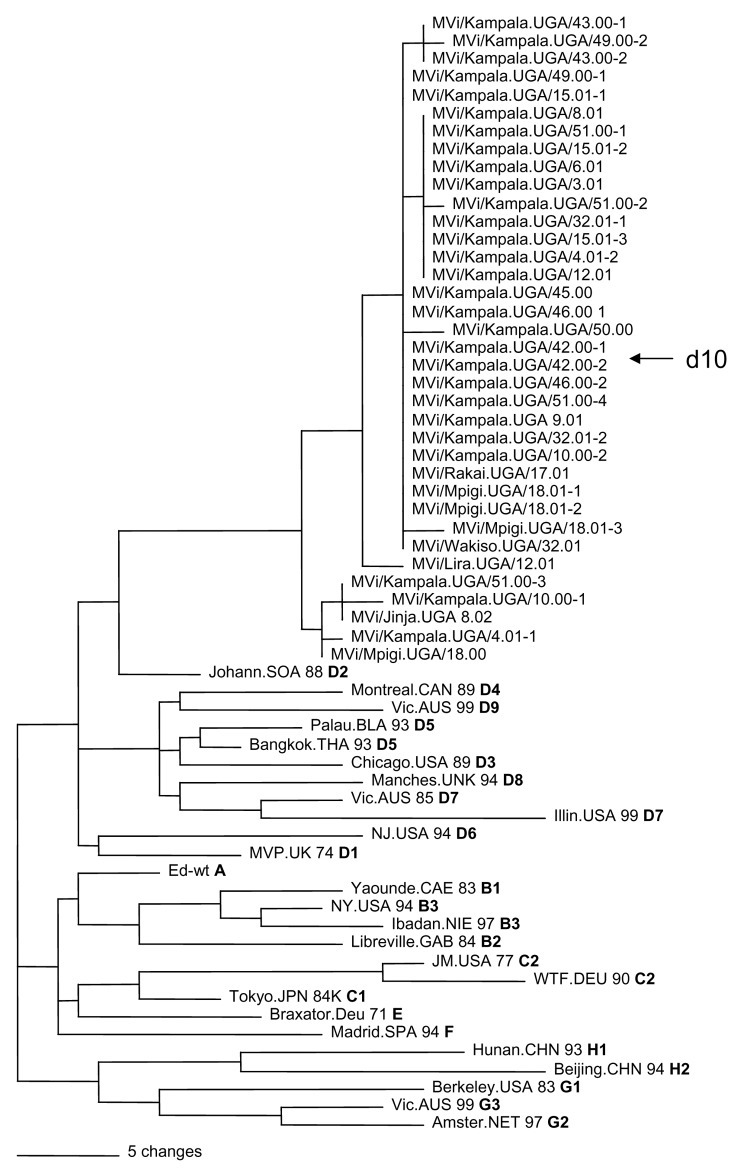
Phylogenetic analysis of sequences of nucleoprotein genes (450 nucleotides) of wildtype measles viruses isolated in Uganda during 2000–2002. The unrooted tree shows sequences from Ugandan viruses compared with World Health Organization reference strains for each genotype. Genotype designation is in bold.

**Figure 3:**
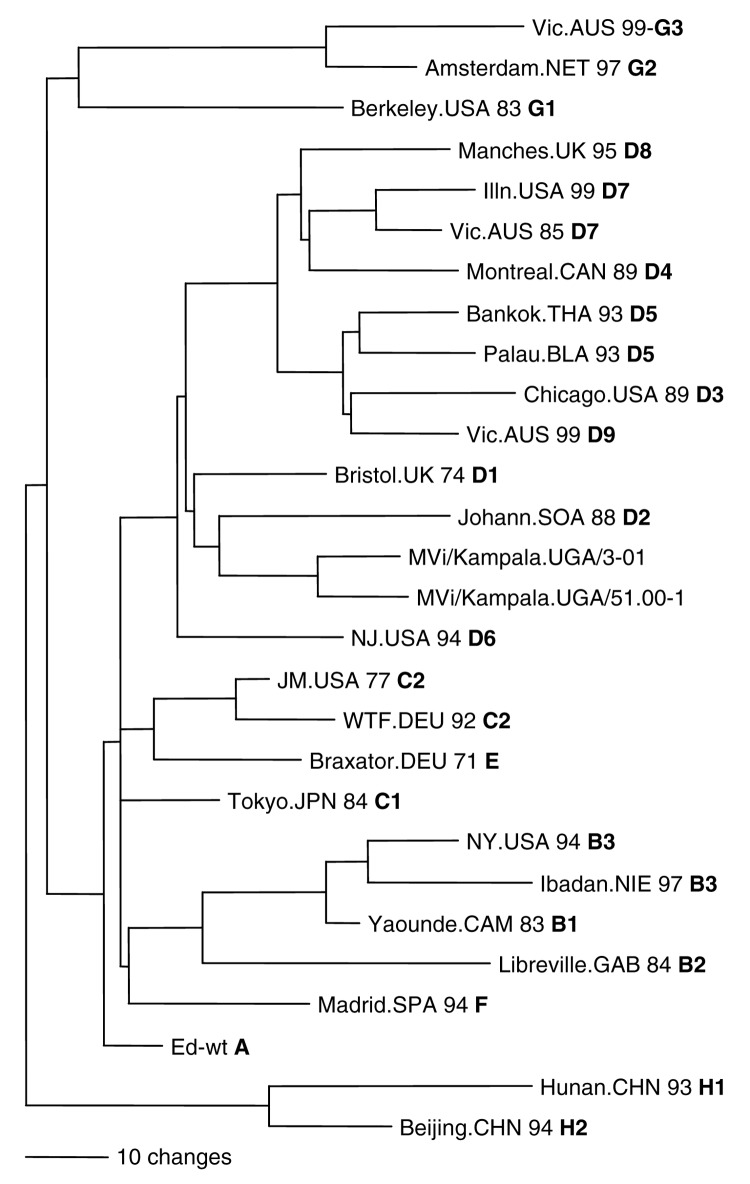
Phylogenetic analysis of sequences of hemagglutinin genes of wildtype measles viruses isolated in Uganda during 2000–2002. The unrooted tree shows sequences from the Ugandan viruses compared with World Health Organization reference strains for each genotype. Genotype designation is in bold.

Sequence data from this report are available from GenBank under accession nos. AY923185–AY923212 and AY923213–AY92321. All sequences were derived from viral isolates that were passaged once in B95a cells. The region of the N gene that is routinely sequenced for genotyping is not affected by tissue culture passage (*5**,**6*). Analyses of the sequences of the N gene showed that the Ugandan isolates were all closely related and composed of 2 clusters (Figure 2 and Table 2). Most of the Ugandan sequences were in a single cluster in which the sequences differed by <6 nt (1.3%). Within this large cluster, 2 groups were composed of 14 and 9 viruses each that had identical N-gene sequences. Nucleotide divergence within the smaller cluster was 0.6%, and overall, Ugandan viruses differed from each other by 0–11 nt (0%–2.4%) in the N gene. Viruses from Mpigi, Kampala, and Jinja were found in both clusters, and viruses from Lira, Wakiso, and Rakai were part of the larger cluster. The sequence of the entire coding region of the H gene was obtained for 2 of the Ugandan viruses (MVi/Kampala.UGA/51.00-1 and MVi/Kampala.UGA/3.01). The Ugandan H-gene sequences differed by 21 nt (1.1%) and 6 predicted amino acids (Figure 3).

**Table 2:** Percentage genetic distance between wildtype measles viruses from Uganda and World Health Organization reference strains

Genotype*	Nucleoprotein gene (range)†	Hemagglutinin gene‡
A	5.1–6.2	2.2
B1	6.8–8.2	3.2
B2	6.4–7.7	4.0
B3-NY USA/94	6.6–7.9	3.8
C1	5.1–5.9	2.3
C2-Erlangen Due/90	8.6–9.9	3.1
D1	4.4–5.7	2.2
D2	3.1–4.4	2.6
D3	4.8–6.4	3.5
D4	5.5–6.4	3.1
D5-Palau BLA/93	4.8–6.4	3.1
D6	5.7–7.0	2.4
D7-Illinois USA/99	7.7–9.0	3.0
D8	5.3–6.6	3.0
D9	5.7–7.3	3.0
E	5.5–6.8	2.9
F	6.2–7.5	3.0
G1	7.7–9.0	4.2
G2	7.2–8.6	4.6
G3	6.8–8.2	5.3
H1	7.9–8.8	5.5
H2	7.3–9.0	4.6

*One reference sequence was used for each comparison. For a genotype with >1 reference sequence, the indicated sequence was used for comparison. †Range of percentage nucleotide differences between all Uganda sequences and the reference sequences for the nucleoprotein gene. ‡Since we had only 2 Ugandan hemagglutinin gene sequences, the lowest percentage difference is shown. However, both percentages did not vary by >0.1%.

Phylogenetic analyses based on both N and H gene sequences showed that the Ugandan viruses were members of clade D. However, the sequences of the Ugandan viruses were not closely related to the sequences of any WHO reference sequences that represented the 22 currently recognized genotypes. The Ugandan sequences were closest to the sequence of the genotype D2 reference strain. The minimum nucleotide divergence between the Ugandan viruses and the D2 reference strain was 3.1% for the N gene and 2.6% for the H gene (Table 2).

Bootstrap analysis of Ugandan H-gene sequences and the WHO reference sequences showed 100% confidence in the group containing Ugandan viruses. When N-gene sequences were compared with WHO reference sequences and contemporary genotype D2 (*13*) and D4 (*14**,**15*) sequences, bootstrap support for the Ugandan branch was 100% (data not shown). Therefore, based on the current WHO recommendations for designating a new genotype (*7**,**8**,**16*), the Ugandan viruses should be considered as a new genotype of measles. The proposed genotype that includes the Ugandan viruses is genotype d10. Mvi/Kampala.UGA/51.00-1 (GenBank accession nos. N:AY923185, H:AY923213) was chosen as the reference strain for d10 because it represents most isolates and grows to high titers in cell culture.

## Discussion

This is the first report of the genetic characterization of wildtype measles from Uganda and the second that describes characterization of viruses from eastern Africa. Although this study was successful, it highlights some of the difficulties in performing virologic surveillance in developing countries. The relatively low rate of virus isolation in this study was surprising considering that all the specimens were collected within 7 days of rash onset. However, many specimens were not collected properly, and many arrived at the laboratory in poor condition because of inadequate transportation. Better training and adequate specimen-collection equipment and supplies could prevent some of these problems. In addition, many of the specimens were contaminated, and the filtration step for removing the contaminants reduced the viral titer. Specimens that do not require reverse cold chain, such as blood spots dried onto filter paper, will improve the efficiency of virologic surveillance in countries such as Uganda.

Our results show that the percentage sequence divergence between the N and H gene sequence of the Ugandan virus isolates and the sequences of the reference strains exceeds the recommended threshold for designation of a new measles genotype, which is 2.5% and 2.0% minimum nucleotide divergence for the COOH region of the N and H genes, respectively (*7**,**8**,**16*). Phylogenetic analyses group these viruses in clade D. The results also showed that the Ugandan viruses had sequences that were unique among all previously characterized wildtype measles viruses and constitute a new genotype, which we propose to be genotype d10. All viruses isolated over the 2-year period from 6 districts in Uganda belonged to this single, proposed new genotype. Therefore, d10 should be considered the endemic genotype in Uganda. The degree of genetic relatedness observed for the Ugandan viruses is surprising since these viruses were isolated during a period when measles was widespread in the country. Previous genetic analyses of wildtype measles viruses from countries with endemic measles have shown more genetic heterogeneity within a genotype, which suggested the presence of multiple chains of transmission (*12**,**17**,**18*).

The Ugandan viruses have a unique genetic signature that clearly distinguishes them from other African viruses. During the time when d10 viruses were isolated in Uganda, genotype D4 viruses were circulating in nearby Kenya (*15*) and genotypes D4 and D8 were detected in Ethiopia (*14*). Genotype B3, the most prevalent genotype in western Africa, has also been detected in Sudan and Democratic Republic of Congo, which are north and west, respectively, of Uganda (*18**–**23*). D2 and D4 are the most frequently detected genotypes in southern Africa (*24*), while genotype C2 viruses were detected in northern Africa in Morocco (*25*). Baseline virologic surveillance has not been conducted in Tanzania, Rwanda, or Burundi, the countries that border Uganda to the south. Therefore, the exact geographic distribution of genotype d10 viruses is unknown.

The purpose of virologic surveillance is to establish the transmission pathways of measles virus. Both Uganda and Kenya have initiated accelerated measles control activities and successfully completed a baseline survey of viral genotypes. Molecular epidemiologic techniques will now be very useful in monitoring the transmission pathways in eastern Africa, contribute to the development of effective measles control strategies, and document the success of the measles vaccination program.
